# Highly stable and antifungal properties on the oilseed rape of Cu_3_(MoO_4_)_2_(OH)_2_ nanoflakes prepared by simple aqueous precipitation

**DOI:** 10.1038/s41598-024-53612-0

**Published:** 2024-03-04

**Authors:** Zhao Xu, Xu Lisha, Liu Yi, Mei Yunjun, Chen Luocheng, Zheng Anqi, Yin Kuibo, Xiao Xiaolu, Li Shaozhen, Sun Xuecheng, Zhang Yifu

**Affiliations:** 1 School of Electrical and Electronic Engineering, Wuhan Polytechnic University, Wuhan, 430023 Hubei China; 2 School of Physics, Hubei University, Wuhan, 430062 China; 3 School of Chemical and Environmental Engineering, Wuhan Polytechnic University, Wuhan, 430023 Hubei China; 4 Hubei Sino-Australian Nano Material Technology Co., Ltd., Guangshui, 432700 China; 5 SEU-FEI Nano-Pico Center, Key Laboratory of MEMS of Ministry of Education, Southeast University, Nanjing, 210096 People’s Republic of China; 6 Key Laboratory of Biology and Genetic Improvement of Oil Crops of Ministry of Agriculture and Rural Affairs, Oil Crops Research Institute of the Chinese Academy of Agricultural Sciences, Wuhan, 430062 Hubei China; 7 Micro-Elements Research Center, College of Resource and Environment, Huazhong Agricultural University, Wuhan, 430070 China; 8 Department of Chemistry, School of Chemical Engineering, Dalian University of Technology, Dalian, 116024 China

**Keywords:** Plant sciences, Materials science, Nanoscience and technology

## Abstract

In the last few decades, nanoparticles have been a prominent topic in various fields, particularly in agriculture, due to their unique physicochemical properties. Herein, molybdenum copper lindgrenite Cu_3_(MoO_4_)_2_(OH)_2_ (CM) nanoflakes (NFs) are synthesized by a one-step reaction involving *α*-MoO_3_ and CuCO_3_⋅Cu(OH)_2_⋅*x*H_2_O solution at low temperature for large scale industrial production and developed as an effective antifungal agent for the oilseed rape. This synthetic method demonstrates great potential for industrial applications. Infrared spectroscopy and X-ray diffraction (XRD) results reveal that CM samples exhibit a pure monoclinic structure. TG and DSC results show the thermal stable properties. It can undergo a phase transition form copper molybdate (Cu_3_Mo_2_O_9_) at about 300 °C. Then Cu_3_Mo_2_O_9_ nanoparticles decompose into at CuO and MoO_3_ at 791 °C. The morphology of CM powder is mainly composed of uniformly distributed parallelogram-shaped nanoflakes with an average thickness of about 30 nm. Moreover, the binding energy of CM NFs is measured to be 2.8 eV. To assess the antifungal properties of these materials, both laboratory and outdoor experiments are conducted. In the pour plate test, the minimum inhibitory concentration (MIC) of CM NFs against *Sclerotinia sclerotiorum (S. sclerotiorum)* is determined to be 100 ppm, and the zone of inhibiting *S. sclerotiorum* is 14 mm. When the concentration is above 100 nm, the change rate of the hyphae circle slows down a little and begins to decrease until to 200 ppm. According to the aforementioned findings, the antifungal effects of a nano CM NFs solution are assessed at different concentrations (0 ppm (clear water), 40 ppm, and 80 ppm) on the growth of oilseed rape in an outdoor setting. The results indicate that the application of CM NFs led to significant inhibition of *S. sclerotiorum*. Specifically, when the nano CM solution was sprayed once at the initial flowering stage at a concentration of 80 ppm, *S. sclerotiorum* growth was inhibited by approximately 34%. Similarly, when the solution was sprayed once at the initial flowering stage and once at the rape pod stage, using a concentration of 40 ppm, a similar level of inhibition was achieved. These outcomes show that CM NFs possess the ability to bind with more metal ions due to their larger specific surface area. Additionally, their semiconductor physical properties enable the generation of reactive oxygen species (ROS). Therefore, CM NFs hold great potential for widespread application in antifungal products.

## Introduction

Proper cultivation practices for oilseed rape, beans, and other major crops are crucial for ensuring food and oil security. Especially in recent years, the occurrence of extreme events, such as the outbreak of the Covid-19 epidemic, extreme weather conditions, and pest outbreaks, has become more frequent. These factors, to some extent, have disrupted the international trade of oilseed crops among different countries. Furthermore, a variety of diseases, such as *Sclerotinia sclerotiorum* (*S. sclerotiorum*), *downy mildew* and *bacterial wilt,* have significantly impacted the yield of oilseed crops. It was established that molybdenum was an essential trace element for plants in 1939. Since then, scientists have paid much attention to molybdate compounds for their anti-viral properties^1–3^. In 2014, Deepak pioneered the utilization of a polyaniline tungsten-molybdenum phosphate system for the separation of toxic metals from water^4^. Furthermore, the same research team synthesized and characterized stannous molybdate and discussed its antimicrobial properties^5^. Numerous other molybdate systems have been studied for their antimicrobial properties^6–10^.

As a rare natural mineral material, Cu_3_(MoO_4_)_2_(OH)_2_ (CM) has received great attention due to its excellent magnetic, photocatalytic, and electrochemical properties^11^. CM thin films can be used as photocatalysts for the photoconversion of CO_2_ into valuable compounds^12^. Copper molybdate material has a major inhibitory and bactericidal effect against *Escherichia coli* (*E. coli*)^13–15^, *Staphylococcus aureus*, and *Salmonella*^15^. A plant disease, *S. sclerotiorum*, exists in many important crops^16,17^. It causes more than 10% yield loss in China^18^. To prevent harm to the environment, livestock, and human health caused by antifungal pesticides^19^, the development of metal or metal oxide nanoparticles^20^ has emerged as a frontier area of antifungal research. Until now, there have been limited reports on the resistance of CM to outdoor fungal infections in oilseed rape. It is well known that some metal silver (Ag), copper (Cu), zinc (Zn), and Titanium (Ti) exhibit strong antimicrobial activity^21^. Several compounds, such as CuSO_4_, are also used to control algal and fungal proliferation^22,23^. Considering the factors mentioned above, the study on nano CM powder paves a feasible approach to inhibit *S. sclerotiorum* infections and thus improve the yield of oilseed rape.

Generally, several methods were used to prepare CM nanoparticles^4,5,10^. The nano-characteristics of Cu_3_(MoO_4_)_2_(OH)_2_ is a topic worthy of further study, and related research results have been reported in the literature. Rahimi et al*.* used chemical precipitation and hydrothermal methods to realize the simple and controllable synthesis of Cu_3_(MoO_4_)_2_(OH)_2_ nanoparticles and carried out structural characterization^24^. Liu et al*.* prepared Cu_3_(MoO_4_)_2_(OH)_2_ nanorods employing electro-chemistry-assisted laser ablation in liquid (ECLAL)^25^. Swain et al*.* synthesized Cu_3_(MoO_4_)_2_(OH)_2_ nanoflowers by water precipitation process at room temperature and transformed the product into Cu_3_Mo_2_O_9_ after thermal annealing^11^. Xia et al*.*^26^ used hydrothermal and sintering methods to prepare monodisperse microparticles. Curved nanoflakes were produced by Xu et al*.*^27^ using a liquid–solid reaction in a hierarchical architecture microreactor. Jiang et al*.*^28^ developed an aqueous precipitation approach at 60 °C to obtain nano-sized tabular structures using Cu(NO_3_)_2_·6H_2_O, Na_2_MoO_4_·2H_2_O and NaOH precursors. Hollow and prickly sphere-like nanostructures were constructed by Yan et al*.*^29^ utilizing hydrothermal and solvothermal strategies. However, some of these methods maybe have some own shortcomings, for example, organic chemicals which have environmental limitations, or complex instrumentation, in the lab and low yields. All of them result in low yield, and limitation of the application of nano Cu_3_(MoO_4_)_2_(OH)_2_. Therefore, to study the antifungal properties of Cu_3_(MoO_4_)_2_(OH)_2_, the initial step is to realize the industrial preparation of nano Cu_3_(MoO_4_)_2_(OH)_2_.

**Figure 1:**
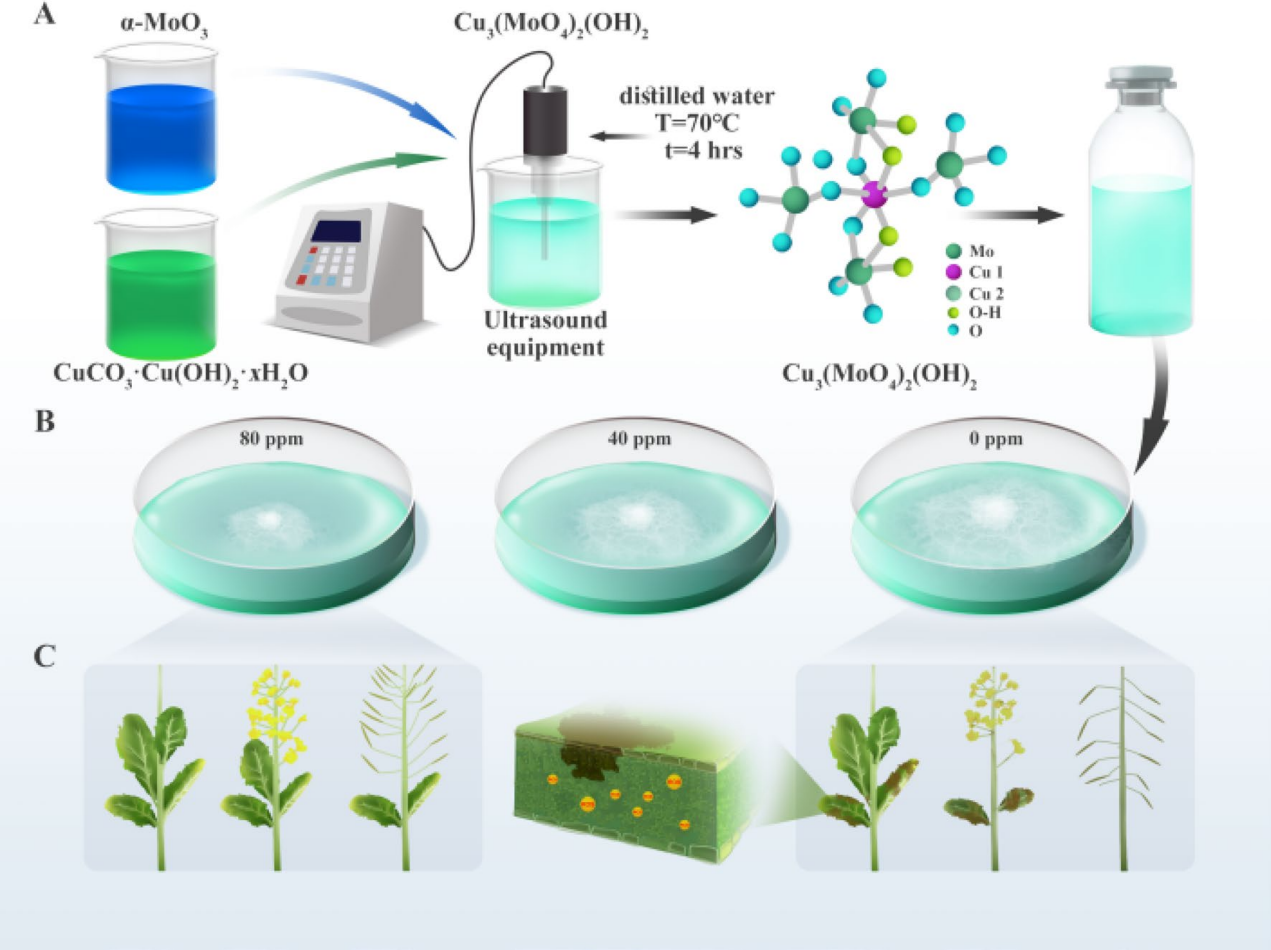
Schematic illustration of the process of simple aqueous precipitation (**A**) and antifungal application (**B**, **C**) of the Cu_3_(MoO_4_)_2_(OH)_2_ nanoflakes.

In this study, we design molybdenum copper lindgrenite Cu_3_(MoO_4_)_2_(OH)_2_ nanoflakes using aqueous precipitation, aiming for large-scale industrial production as illustrated in Fig. 1A. The Cu_3_(MoO_4_)_2_(OH)_2_ powder exhibits a mainly 30 nm in thickness and a parallelogram shape. Further studies on the antifungal characteristics of Cu_3_(MoO_4_)_2_(OH)_2_ show a significant inhibition against fungal growth outdoor. The minimum inhibitory concentration (MIC) is 100 ppm, and the minimum fungicidal concentration (MFC) is 1000 ppm. Outdoor planting trials show that spraying once with an 80 ppm concentration at the initial flowering stage of rapeseed, or spraying with a concentration of 40 ppm once at the initial flowering stage and once at the rape pod stage. It can reduce the incidence of *S. sclerotiorum* by more than 34% throughout the growth cycle of rapeseed. On the one hand, the primary mechanism of *S. sclerotiorum* inhibition is attributed to the effect of copper ions. It is speculated that CM NFs have the effect of enhancing the immune response of rapeseed, which could be the potential reason for the reduction of incidence throughout the growth cycle of rapeseed after spraying CM NFs once or twice. On the other hand, increasing the surface area of nanoflakes results in the enhancement of the contacting probability between cupric ions and fungal cells. The semiconductive physical properties of CM NFs raise the emergence of reactive oxygen species (ROS), which further enhances the antifungal effects.

## Experimental

### Materials and methods

A low-cost, industrialized method for preparing Cu_3_(MoO_4_)_2_(OH)_2_ was designed. Raw materials are nano molybdenum trioxide (purity, *α*-MoO_3_, 99.9%) produced by Zhongao NanoTech Co., Ltd, and industrial copper carbonate (purity, CuCO_3_⋅Cu(OH)_2_⋅*x*H_2_O, 99.9%) product from Shanghai Runtai Pharmaceutical Technology Co., Ltd.

The sample of Cu_3_(MoO_4_)_2_(OH)_2_ was prepared by an aqueous solution procedure, as depicted in Fig. 1A. The raw materials were high-purity copper carbonate (99.8%) and α-MoO_3_, where the molar ratio of Cu and Mo in the raw materials was 7:8 by self-made equipment. The steps for the preparation of nano-basic copper molybdate were as follows: firstly, the mixture of high-purity copper carbonate and α-MoO_3_ was heated in a muffle furnace to 70 °C for 2 h, and then heated to 120 °C for the other 2 h, to produce a nano-molybdate copper powder. Secondly, the non-ionized water (free of alkali metal ions) and the dispersant ethylene glycol (1%) were added into the ultrasonic reactor at a mass ratio of 99:1 and mixed with ultrasonic shock treatment for 2 h. Then, the powder generated by the reaction was cooled; the powder was put into the above ultrasonic reactor with the pre-treated non-ionized water with the mass ratio of 1:20, and then subjected to ultrasonic shock treatment for 4 h. Finally, the aqueous solution stood for 3 h and the nano-basic copper molybdate was prepared.

Measurements of the crystal structure of the samples were performed on a ‘Rigaku UltimaIV’ X-ray diffraction apparatus (XRD, 285 mm 3 kW and Cu K*α*1). The thermal behaviors were investigated by Simultaneous Thermal Analyzer (STA 449F5, RT ~ 1600 °C). The morphology and composition of the material were carried out on Zeiss Gemini SEM300. Ultra-high-resolution transmission electron microscope (HRTEM, Titan 80-300) with both spherical aberration correction and monochromator (spatial resolution 80 pm, EELS energy resolution 0.3 eV) were used to get the microscopic images and detailed material structure information. The testing of the valence of the elements was completed by an X-ray photoelectron spectroscopy (XPS, ARL9800XP+). The banding energies of CMO NPs were carried out by Ultraviolet Photoelectron Spectroscopy (UPS) with an Axis Supra of KRATOS.

### Procedure of the inhibition test

In the antifungal performance experiment, the fungal strain used in the test was *S. sclerotiorum* (CGMCC 3.7083, the Institute of Microbiology of the Chinese Academy of Sciences). The experimental procedures are as follows:

Firstly, use the complete preparation method^30^ to configure all potato dextrose agar (PDA) solid media for fungus culture; secondly, cool the high-temperature steam sterilized PDA solid to 50 °C, and pour it into a plate on a sterile ultra-clean workbench (the diameter of the circular plate petri dish is 9 cm). Then add the pre-prepared sterile copper molybdate solution (10% stock solution concentration) to the PDA solid medium to reach the preset concentrations of the experiment to make a series of gradient plates containing copper molybdate. Use an inoculating loop to scrape the hyphae from the purchased *S. sclerotiorum*—containing PDA medium, inoculate the hyphae on a plate containing fresh PDA medium, and culture it at 28 °C for 4 days (when mycelia cover the plate) to complete the first activation.

Excise a 5-mm-diameter disc from each aforementioned 4-day-old PDA culture with a sterile puncher (5 mm inner diameter), and inoculate the *S. sclerotiorum*—containing a PDA disc on another aseptic PDA plate. Each plate is only inoculated with a 5-mm-diameterhyphae-containing PDA disc, cultivated at 28 °C for 4 days to complete the second activation, and instantly used for subsequent antifungal experiments.

Afterwards, excise a 5-mm-diameter disc from each second activation culture with a sterile puncher (5 mm inner diameter), and inoculate the 5-mm-diameter *S. sclerotiorum*—containing a PDA disc on a PDA plate (control group) and copper molybdate plates with different concentrations (experimental group). Both the control group and the series concentration experimental group have three repetitions. The inoculated plates are cultured in an incubator at 28 °C for 36 h. Finally, measure the growth radius of mycelia along the inoculated agar disk in the control group and the experimental group, and calculate the inhibitory effect.

### Spraying methods

Spraying CM NFs aqueous solution onto the surface of the rape leaves was chosen to be carried out under low light intensity and minimal wind conditions. This was primarily to minimize evaporation and wind-induced drying. As a consequence, CM NFs aqueous solution turned into particles on the leaf surface, which subsequently detached from the leaf surface within a short period of time. The nano CM aqueous solution was diluted to the concentrations of 0 ppm (water), 40 ppm and 80 ppm, and manually sprayed onto the plants.

### Test specifications for *S. sclerotiorum* in oilrape

All measurments about the plants are performed by institute of the Chinese Academy of Agricultural Sciences Collection of plant material. It is complied with relevant institutional, national, and international guidelines and legislation from China. In the investigation of rape *S. sclerotiorum*, disease severity was calculated statistically based on disease incidence and disease index following the agricultural trade standard (China MoAotPsRo (2011), Rules for investigation and forecast technology of rape sclerotiniose).

### Ethical approval

The experiments conducted at Wuhan Polytechnic University are solely related to antifungal research.

## Results and discussion

### Synthesis of Cu_3_(MoO_4_)_2_(OH)_2_ NFs

Figure 1 shows the schematic illustration of the simple aqueous precipitation method (A) and antifungal application (B and C) of the Cu_3_(MoO_4_)_2_(OH)_2_ NFs. The industrial aqueous solution offers three advantages, as shown in Fig. 1A: (1) it is a chemically “simple and clean” synthesis because of the simple starting materials, no catalyst requirement, and reduced unwanted byproduct formation etc*.*; (2) an ambient environment is used without requiring extreme temperature and pressure conditions; (3) it enables high yield (15 kg per each time) and low solubility of CM NFs. Therefore, CM NFs were obtained by the industrial aqueous solution at low temperature by a one-step reaction. Then the killing effect of these nanoflakes on *S. sclerotiorum* and the mechanism of action are presented in Fig. 1B,C. This strategy has many advantages: (1) CM nanoparticles have excellent ROS-generating ability and can effectively reduce oxidative stress in microorganisms; (2) CM nanoparticles have good biological safety as they contain an essential trace element for plants. By exploring the mechanism behind the inhibitory effect of CM nanosolutions on *S. sclerotiorum* infection, we can expand the applications of CM in plant protection. A critical step is to study the structure and composition of CM.

**Figure 2:**
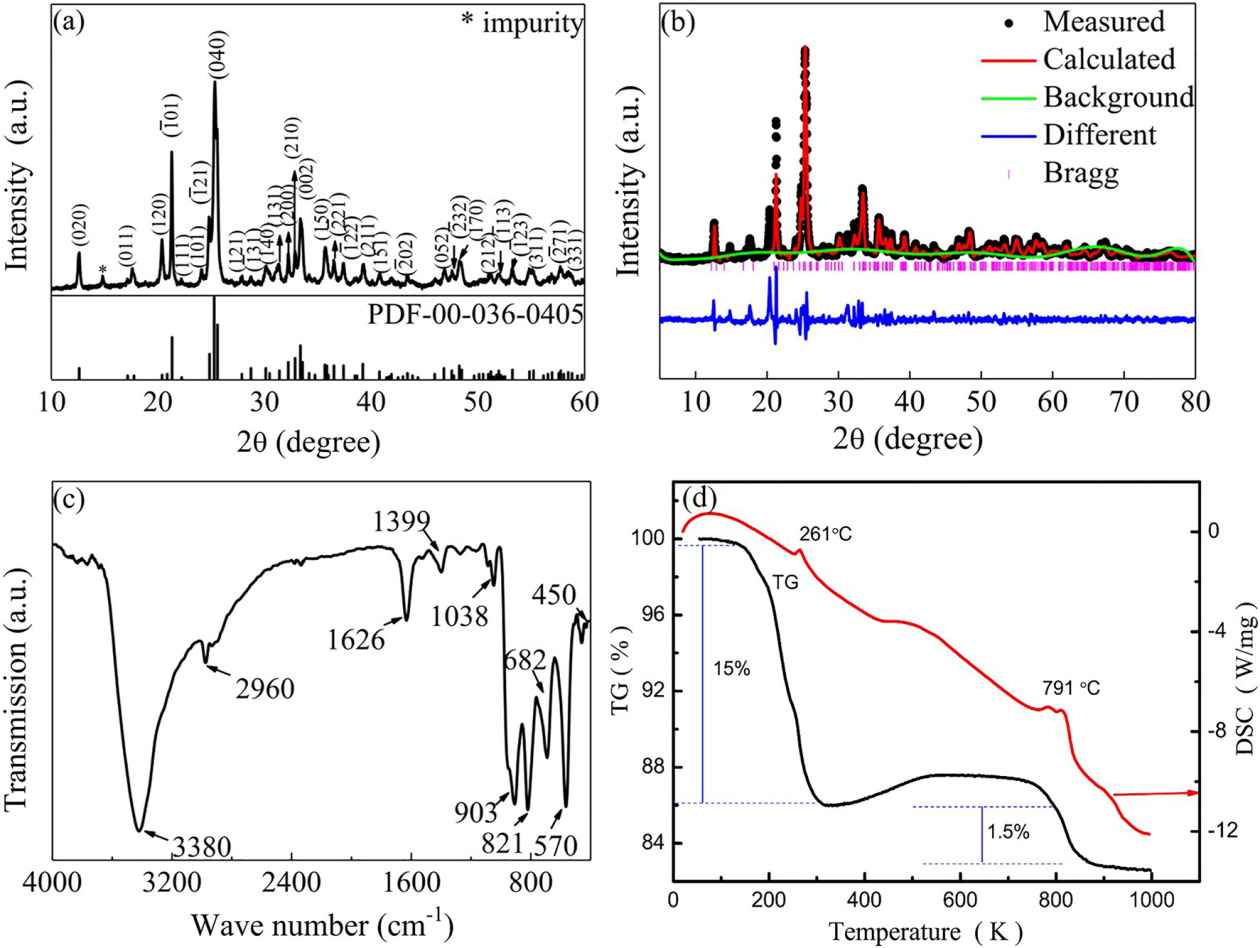
XRD powder diffraction patterns of Cu_3_(MoO_4_)_2_(OH)_2_ (**a**) and patterns calculated by Gasas Rietveld method (**b**); FTIR spectrum of synthesized Cu_3_(MoO_4_)_2_(OH)_2_ nanoflakes (**c**); TG and DSC curves of Cu_3_(MoO_4_)_2_(OH)_2_ powder (**d**).

X-ray diffraction analysis and the Joint Committee on Powder Diffraction Standards (JCPDS) card number 00-036-0405 were utilized to demonstrate the crystalline phase and purity of the freshly prepared materials, as shown in Fig. 2a. All the diffraction peaks are well-matched with the standard cards. It behaves as the space group of *P*2_1_*/n.* The sample has good crystallinity, and it shows Cu_3_(MoO_4_)_2_(OH)_2_ crystalline phase, with a minor amount of MoO_3_ powder. Calculated by the Gasas Rietveld method in Fig. 2b, the cell parameters of the sample are *a* = 5.397750 Å, *b* = 14.009352 Å, *c* = 5.606040 Å, Chi^2^ = 2.775, *R*_*wp*_ = 4.88%, and *R*_*p*_ = 3.46%.

To confirm the chemical structure of synthesized CM NFs, the FT-IR spectrum is analyzed. Figure 2c shows several distinct bands at 450, 570, 682, 821, 903, 1038, 1399, 1626, 2960 and 3380 cm^−1^. The bands that appeared from 300 to 500 cm^−1^ are due to the O–Mo–O symmetric and anti-symmetric bending vibrations (Fig. 2c). The bands that occurred at 682 cm^−1^ and 570 cm^−1^ are attributed to the *v*_3_ MoO_4_ vibration band. Furthermore, the bands visible from 700 to 1000 cm^−1^ correspond to the MoO symmetric and anti-symmetric stretching vibrations (*ν*_1_ and *ν*_3_ modes) within the MoO_4_ tetrahedra. The peaks at 928 and 959 cm−^−1^ represent the symmetric stretching vibration peaks of the Mo=O bond. The strong bands visible at 821 cm^−1^ and 903 cm^−1^ are due to the *v*_3_ MoO_4_ vibration modes. The peaks shown at 1038 cm^−1^, 1399 cm^−1^, 1626 cm^−1^, 2960 cm^−1^ and 3380 cm^−1^ are ascribed to the infrared vibration of OH groups and H–O···H (hydrogen bonding), respectively. Although in our results all absorption bands were slightly shifted compared with the original *v*_1_ and *v*_3_ MoO_4_ bands, they were consistent with the reported values^11,31–34^. The thermal behavior of Cu_3_(MoO_4_)_2_(OH)_2_ was investigated by TG-DSC in a nitrogen atmosphere at a heating rate of 10 °C/min from 80 to 1000 °C. As shown in Fig. 2d, the total weight loss is 16.5%. The TG curve shows that the weight loss increases comparatively quickly from room temperature to 300 °C on the first step. This stage of mass loss is related to lindgrenite dehydration, which leads to the formation of Cu_3_Mo_2_O_9_ and on the second step, it slightly changes during the remaining time until 791 °C ^11^. The weight loss slowly increased with a weight loss of 1.5%, which is the thermal decomposition of Cu_3_Mo_2_O_9_ into CuO and MoO_3_. All these correspond to the results from two endothermic peaks on the DSC curve. The first one at 261 °C should be ascribed to the hydrate, and the second one at 791 °C means Cu_3_Mo_2_O_9_ depomposition. All are consistent with the previous reports^11,19^.

**Figure 3:**
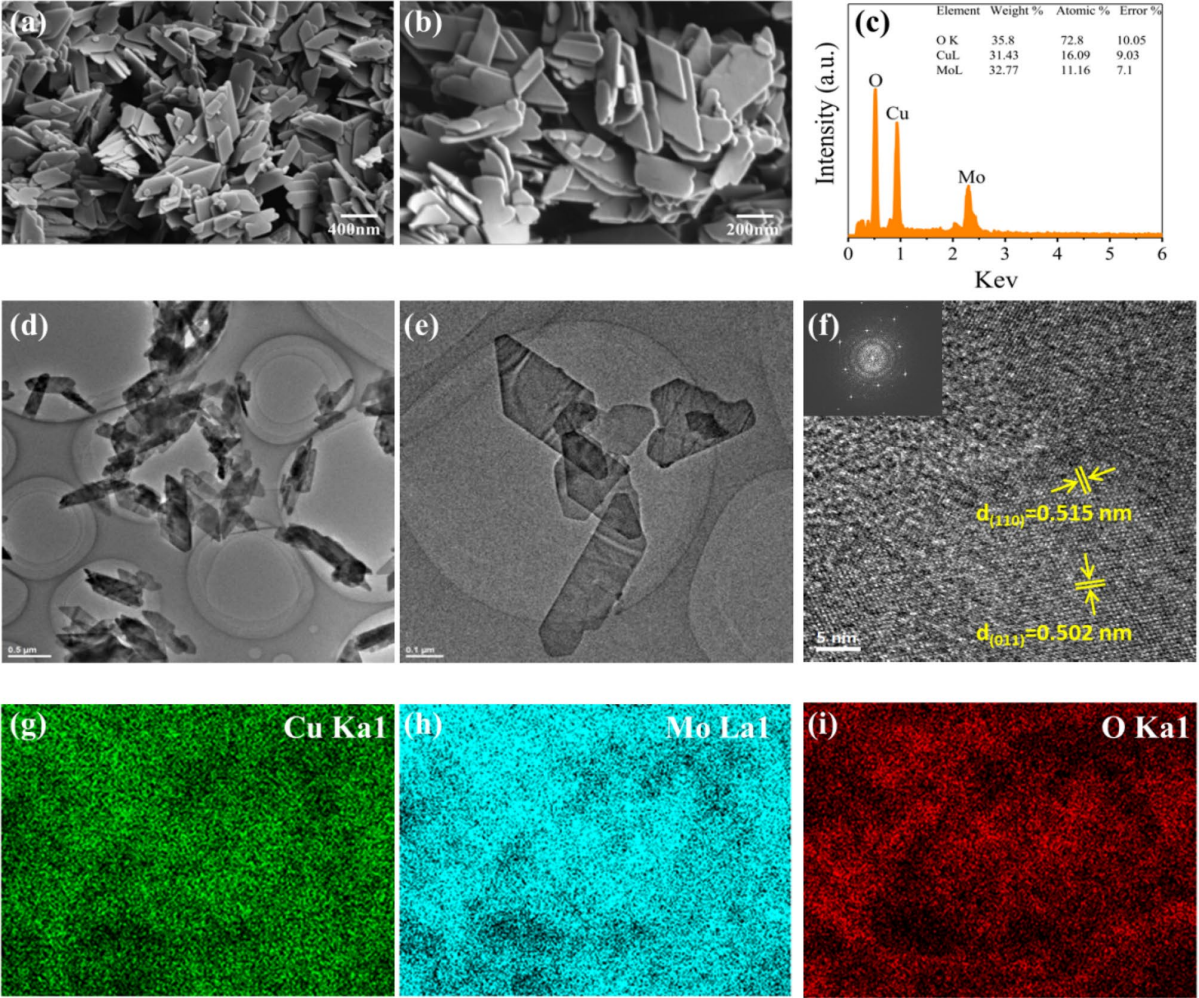
FESEM images (**a**–**c**), EDS mapping (**g**–**i**), and High-resolution TEM images (**d**–**f**) of nano Cu_3_(MoO_4_)_2_(OH)_2_ powder.

The morphologies of Cu_3_(MoO_4_)_2_(OH)_2_ are observed by SEM and TEM. Figure 3a,b show that most of them are parallelogram shapes. The length of the parallelogram is greater than 400 nm, and the width is above 300 nm. The elemental weight percentages and atomic percentages of CM NFs are shown in energy dispersive spectrometer (EDS) images of Fig. 3c (accelerating voltage 20 kV, magnification × 10 k). The atomic ratio between Cu and Mo is 1.5:1. Figure 3g–i demonstrates the consistency of the Cu, Mo, and O components in the synthesized CM NFs, indicating the purity of the product. This consistency confirms the presence of all intended elements and suggests good polycrystalline characteristics in the CM NFs. The shape and structure of samples were further investigated by TEM observations. Parallelogram-shaped nanoflakes with a thickness of about 30 nm exist. The severe aggregation of CM with an average size of about 500 nm is observed in Fig. 3d–f. In high-resolution transmission electron microscopy (HRTEM) results of Fig. 3f, the clear lattice fringe of 0.515 nm and 0.502 nm can be distinctly identified, which corresponds to the (110) and (011) planes of CM, respectively. Compared with reported laboratory-produced CM nanoparticles (rod-like and dot-like)^12,35–37^, the industrially prepared flakes are more homogeneous in size; nano-flake-shaped particles can expose more surface area; thus, they can release more Cu ions. These can improve antifungal properties.

**Figure 4:**
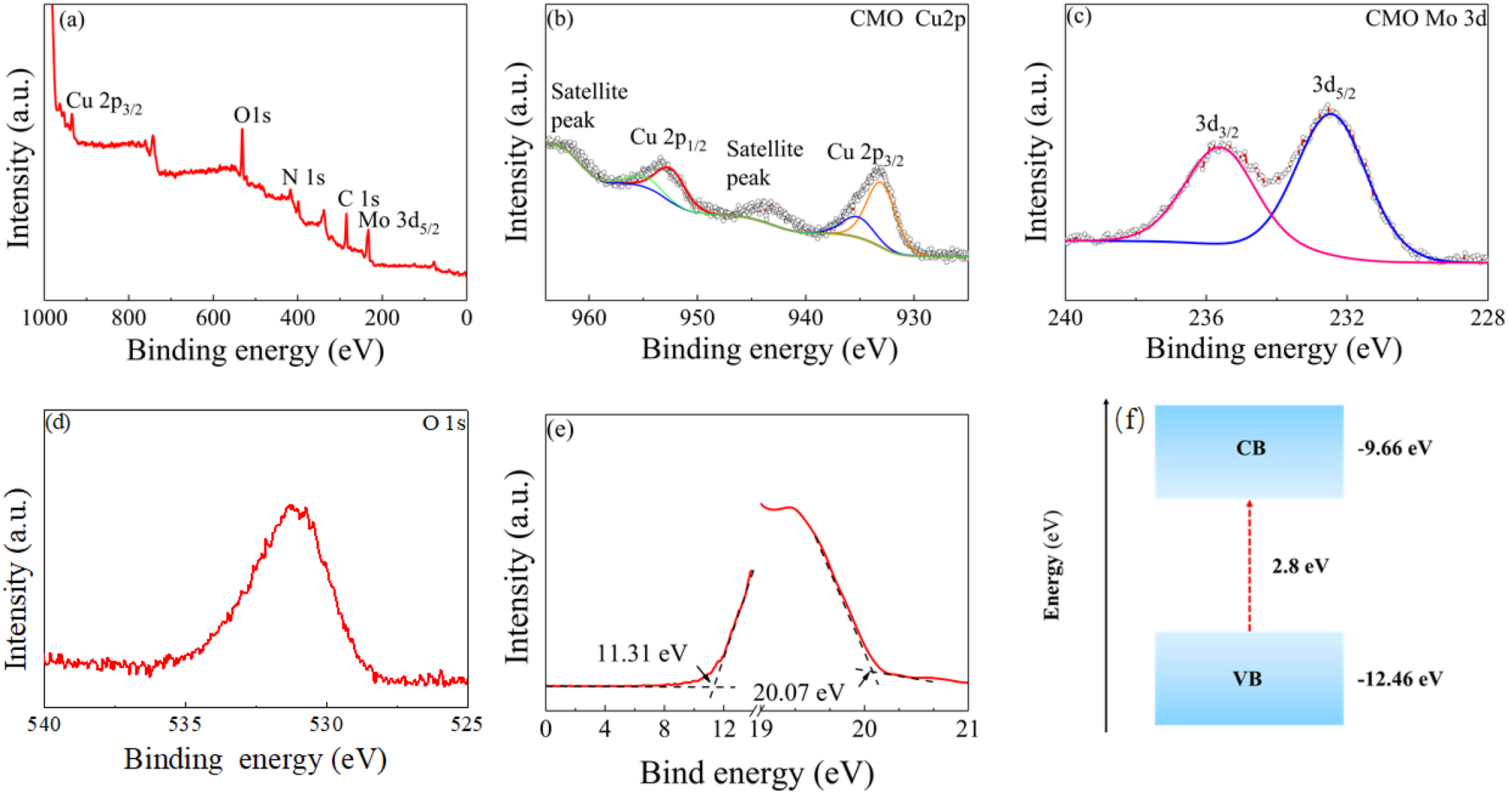
(**a**) Full XPS spectrum of Cu_3_(MoO_4_)_2_(OH)_2_, (**b**) High-resolution Cu 2p, and (**c**) Mo 3d XPS spectra of Cu_3_(MoO_4_)_2_(OH)_2_ and (**d**) O1s XPS spectra of Cu_3_(MoO_4_)_2_(OH)_2;_ (**e**) low binding energy and high binding energy of UPS spectrum and (**f**) schematic energy level diagram of CM NFs.

To better analyze the anti-fungi properties of the materials, X-ray photoelectron spectroscopy (XPS) was employed to analyze the chemical state of Cu_3_(MoO_4_)_2_(OH)_2_ nanoparticles. Figure 4a shows the whole spectrum of Cu_3_(MoO_4_)_2_(OH)_2_. Figure 4b–d show the deconvolution spectra of Cu 2*p*, Mo 3*d* and O1*s*. Two peaks at 933.0 and 953.0 eV are attributed to the binding energy of Cu 2*p*_3/2_ and Cu 2*p*_1/2_, respectively, in Fig. 4b. These signals match the literature values of the Cu^2+^ state. Figure 4c shows two peaks in the Mo 3*d* spectra at 232.2 eV and 235.3 eV, which coincide with the Mo 3*d*_5/2_ and Mo 3*d*_3/2_. It can be seen in Fig. 4c that the oxidation state of molybdenum in Cu_3_(MoO_4_)_2_(OH)_2_ is + 6. In Fig. 4d, the XPS spectrum of O1*s* reveals a single peak at 531.5 eV, representing the presence of metal–oxygen bonds and the absence of other oxygen defects^38^. In summary, the valence states of CM NFs follow the stoichiometric ratio that contains Mo^6+^.

In order to investigate the physical properties of CM NFs, ultraviolet photoelectron spectroscopy (UPS) spectra have been used to characterize the valence band maximum (VBM) of CM NFs, as shown in Fig. 4e–f. It can be observed that the onset energy (*E*_onset_) of CM NFs is 11.3 eV, while the cutoff energy (*E*_cutoff_) is 20.07 eV. The energy reference is set at the Fermi energy. The VBM of CM NFs is determined to be − 12.46 eV. Meanwhile, the work function of CM NFs is − 9.66 eV. Combined with the conduction band minimum (CBM) derived from the VBM and bandgap (i.e., neglecting the exciton binding energy), it can be found that the energy level is 2.8 eV^19^. The results confirm that CM NFs behave semiconductive properties.

### Fungal inhibition effect

**Figure 5:**
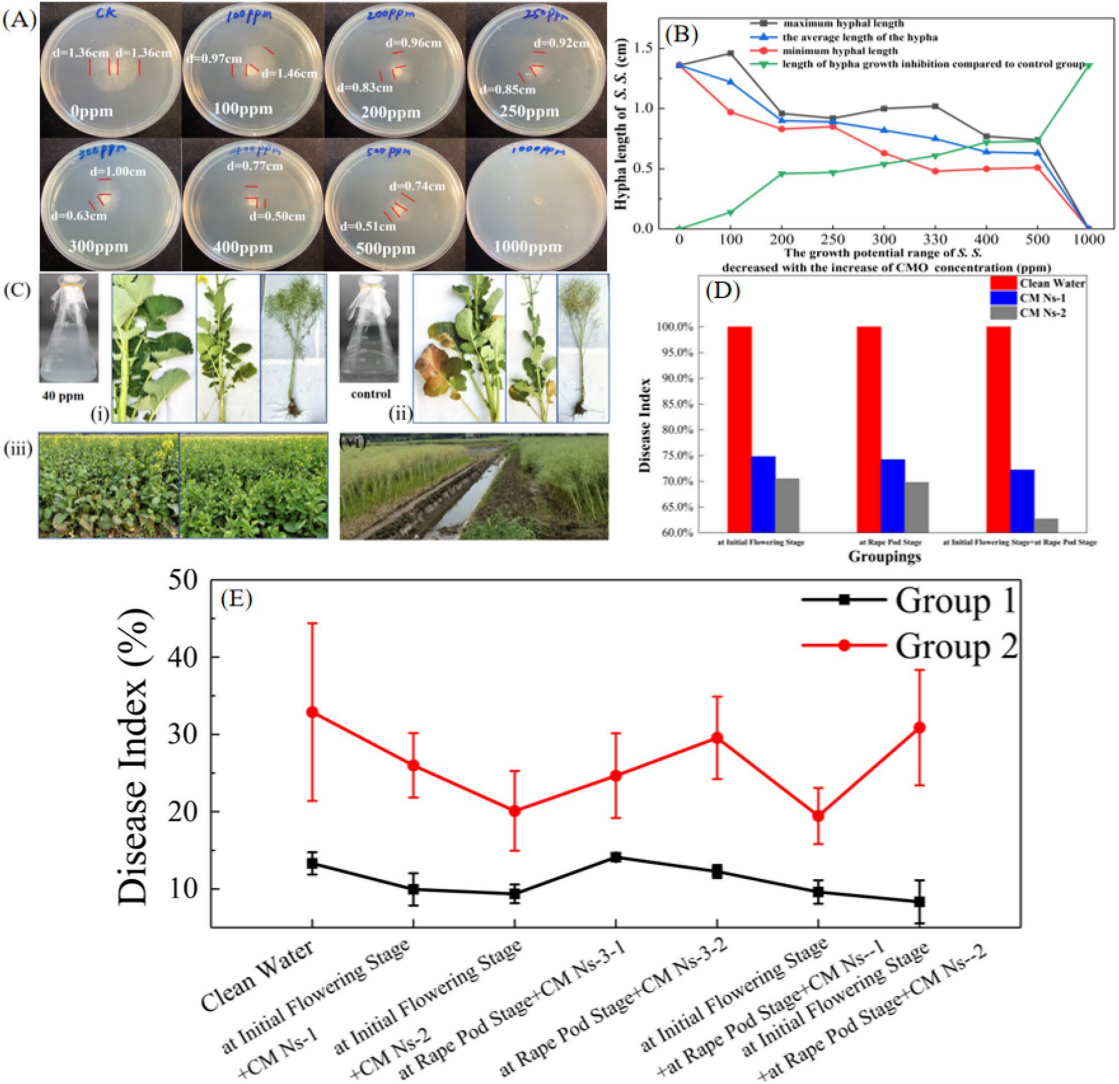
(**A**) The changes of hypha length after Cu_3_(MoO_4_)_2_(OH)_2_ nanoflakes interacted with *S. sclerotiorum* for 36 h at different concentrations. The control group was 0 ppm. (**B**) The relationship between the concentration of Cu_3_(MoO_4_)_2_(OH)_2_ and the inhibition of fungal hyphae; (**C**) Outdoor experimental plants sprayed with 0 ppm nanoflakes. (The brown circular spots are fungal spots); plants with nanoparticles sprayed at 40 ppm at the initial flowering stage, respectively; (**D**) Relationship between spraying concentration and frequency, and incidence of *S. sclerotiorum(s. s.)*; (**E**) Correlation between spraying concentration and frequency, and incidence of *S. sclerotiorum.* Data are presented as mean ± standard deviation (s.d.) with the error bars representing the standard deviation.

After CM NFs of different concentrations interacted with *S. sclerotiorum* for 36 h, the results of fungal inhibition were shown in Fig. 5A–E. The action process of nanosheets on fungi can be divided into three stages. When the concentration equals 100 ppm, the hyphae circle begins to shrink, indicating that 100 ppm is the minimum inhibitory concentration (MIC) of nanosheets against *S. sclerotiorum*. The fungistatic zone at this time is 0.14 cm. As the concentration of nanosheets increased, the mycelial circle shrunk significantly, indicating that the degree of inhibition of fungal growth was positively correlated with the concentration of nanosheets. In the concentration range of 100 ppm to 200 ppm, the rate of change of the hyphae circle was the fastest. As the concentration of nanosheets continued to increase, the change rate of the hyphae circle slowed down; especially in the two concentration intervals of 200–250 ppm and 400–500 ppm, the hyphae circle hardly changed. When the concentration reached 1000 ppm, the hyphae completely disappeared, symbolizing that the fungus had lost the ability to proliferate. This concentration can be considered the minimum fungi concentration (MFC) of nanoflakes.

Among various inorganic nanoparticles, Cu-based nanoparticles are considered to have antifungal effects^39–41^. The role of nano-metal particles in anti-*S. sclerotiorum* is to inhibit the development of *S. sclerotiorum* spores and conidia, eventually leading to the death of fungal hyphae^42^. It is generally believed that molybdenum-containing compounds exhibit better antifungal properties. The pour plate test results have confirmed this point: the active ingredient in the antifungal test is CM NFs. As shown in Fig. 5B, four parameters are the maximum hyphal length *a*, the minimum hyphal length *b*, the average length of the hypha (*a*-*b*)/2 and the anti-fungi zone 1.36-(*a*-*b*)/2, respectively. The growth potential range of *S. sclerotiorum* refers to the growth length of the hypha. The smaller the length of the hypha, the better the inhibition effect on *S. sclerotiorum* by CM NFs. The anti-fungi area changed drastically in the concentration interval of 100–200 ppm. However, the mycelial growth radius changed little in the concentration ranges of 200–250 ppm and 400–500 ppm, which indicated that the strain had a certain tolerance to higher concentrations of CM NFs.

On the CM NFs—containing cultures, the growth of *S. sclerotiorum* mycelium was significantly inhibited. As the concentration of nanoflakes increased from 100 to 1000 ppm, the inhibitory effect on fungal growth was gradually enhanced. Similar phenomena have also been observed in other antifungal tests of nano-metal salts^23,36,43,44^, because metal ions can cause severe damage, including the separation of the cell membranes from the cytoplasm, resulting in cell lysis and impossible germination of sclerotia. Therefore, the antifungal properties of nanoflakes are likely to be related to the semiconducting properties of molybdates inducing the generation of ROS^45^, which will trigger oxidative stress in cells.

### Field planting test

Oilseed rape with a low yield worldwide is mainly due to stem rot resulting from *S. sclerotiorum*^46^. In order to further investigate how CM NFs inhibit *S. sclerotiorum* infection, CM NFs have been sprayed on the oilseed at different growth stages. Zhongyouza 19 was selected as the test sample, and the experiments were carried out in two different experimental sites, referred to as Site 1 and Site 2. At each site, three different concentrations of CM NFs were applied, and each concentration was repeated three times. Therefore, there are nine plots in the experiments, with each plot having an area of 20 m^2^. In general, the concentration of *S. sclerotiorum* outside is lower than that in the plate test. The concentrations of CM nanosolutions are 0 ppm (clear water), 40 ppm and 80 ppm, respectively. Then CM nanosolutions were sprayed onto the oilseed rape plants starting from the initial flowering stage. The information is shown in Table 1.

**Table 1:** The relationship between the spraying stage and spraying concentration.

Processing number	Spraying stage	Spraying concentration (ppm)
1 (CM NS-1)	Initial flowering stage	40
2 (CM NS-2)	Initial flowering stage	80
3 (CM NS-1)	Rape pod stage	40
4 (CM NS-2)	Rape pod stage	80
5 (CM NS-1)	Initial flowering stage and rape pod stage ripening stage	40
6 (CM NS-2)	Initial flowering stage and rape pod stage stage	80
7 (CLEAR WATER)	Control	0

After treatment with CM NFs at 0 ppm and 80 ppm at the initial flowering stage (Fig. 5C), the inoculated leaves outdoors show a much weaker influence from *s. s.* at 80 ppm than the control group treated with water. It is observed that oilseed outside treated with 80 ppm CM NFs grew better and had less infection. The control plants sprayed only with water show severe leaf yellowing and fewer buds, indicating spreading infection of *S. sclerotiorum*. These findings demonstrated to show that CM NFs at 80 ppm validly inhibit *s. s.* infection in oilseed rape plants. In the investigation of rape *Sclerotiniose*, disease severity was calculated statistically based on disease incidence and disease index following the agricultural trade standard (China 2011)^47,48^. CM NFs were diluted to the specified concentrations with water. Then the diluted solution was sprayed onto the foliage of rapeseed at two experimental sites (Site 1 and Site 2), respectively. Figure 5D indicates that after spraying CM Ns-1 (40 ppm) or CM Ns-2 (80 ppm) only once at the initial flowering stage. The disease index of oilseed rape is reduced by 25.2% or 29.5%, respectively, compared to the control group. If spraying CM Ns-1 (40 ppm) or CM Ns-2 (80 ppm) only once at the rape pod stage, the disease index decreases by -6.2% or 7.8%, respectively. And the disease index has worsened, compared with that at the initial flowering stage. When CM Ns-1 (40 ppm) or CM Ns-2 (80 ppm) is sprayed once at the rape pod stage, and once at the initial flowering stage, the disease index is reduced by 27.8% or 37.3% on average.

The results of the disease index at test Site 2 show that it is significantly reduced by 20.9% or 38.8% with spraying CM Ns-1 (40 ppm) or CM Ns-2 (80 ppm) once at the initial flowering stage. The disease index is reduced by 25.0% or 10.1% by spraying CM Ns-1 (40 ppm) or CM Ns-2 (80 ppm) once at the rape pod stage. The disease index is reduced by 6.1% when CM Ns-2 (80 ppm) is sprayed at the initial flowering stage and the rape pod stage. The most considerable reduction of disease index is 40.9% when CM Ns-1 (40 ppm) is sprayed once at the initial flowering stage and once at the rape pod stage.

Based on the results obtained from Site 1 and Site 2, it can be concluded that CM NFs effectively inhibit infection, leading to a decrease in the disease index of oilseed rape overall. Spraying CM Ns-2 (80 ppm) once at the initial flowering stage, the disease index reduces by 34.2% on average. Spraying CM Ns-1 (40 ppm) once at the initial flowering stage and once at the rape pod stage, the disease index decreases by 34.3% on average. So, both treatments of CM NFs could effectively reduce the occurrence of *S. sclerotiorum* disease in rapeseed. The incidence of the disease is reduced. On the contrary, the oilseed rape sprayed with water is more seriously infected by *S. sclerotiorum*.

In Fig. 5E, it can be observed that the inhibition effect from CM Ns-1(40 ppm, lower concentration) sprayed one time at the initial flowering stage and one time at the rape pod stage, is similar to that at CM Ns-2 (80 ppm, high concentration) sprayed only once at initial flowering stage. Their inhibition rates both exceed 34%. Thus, the effect from CM nano solution at low concentrations shows good inhibition on *S. sclerotiorum*.

On the other hand, copper ions may move into the interior of fungal cells or attach to their outer surfaces, leading to apoptosis through protein denaturation and cell membrane disruption and have more active sites to encounter reduction reactions and yield better antioxidant activity^49^. The mechanism of this tolerance to copper ions has also been found in experiments on other fungi^50^; that is, a relatively high concentration of copper ions can stimulate the growth of sclerotia hyphae^51^. The main explanation for this tolerance mechanism is that *S. sclerotiorum* has genes that detoxify ROS and copper ions^52^. The most significant category of radical oxidants (superoxide ion & hydroxyl OH) and non-radicals are the reactive oxygen entities (hydrogen peroxide, organic peroxides). Metal oxide ions are oxidizing species and can trigger ROS production by coupling with hydrogen peroxide or molecular oxygen, contributing to oxidative stress^53^.

As the concentration of nanoflakes increased to 1000 ppm, which reached the tolerance limit of *S. sclerotiorum*, the fungus was eliminated, and the hyphae completely disappeared. Due to the smaller particle size of CM NFs, a larger specific surface area is obtained. This increases the probability of copper ions in contact with fungal cells, thereby enhancing the antifungal effect compared to non-nano copper molybdate. Worth noting that the CM NFs are slightly soluble in water and have little impact on the environment.

In addition, since the control field and the test field are adjacent to each other and CM NFs do not exist in the control field, the test field is subjected to repeated infection by *S. sclerotiorum* from the control field throughout the growth cycle. Since the inherent instability of CM in maintaining a nano-sized structure over an extended period, the enhanced inhibitory effect may also be related to ROS and other factors. Because CM was sprayed on a cloudy day, it is likely that ROS exists to some extent. On the other hand, it is known that CM contains molybdenum, which is also an essential trace element for plant growth. Therefore, it is worthwhile to investigate further whether CM NFs also play a role in enhancing the immunity of rapeseed.

## Conclusions

The molybdenum copper, Cu_3_(MoO_4_)_2_(OH)_2_ or CM, is successfully synthesized at room temperature by an aqueous solution method. The CM powder consists predominantly of uniformly distributed nanoflakes with a thickness of about 30 nm and a parallelogram shape. Further studies on the antifungal characteristics of CM show a significant inhibitory effect. The minimum inhibitory concentration (MIC) is 100 ppm, and the minimum fungal concentration (MFC) is 1000 ppm. Outdoor planting trials demonstrated that a single spray application of 80 ppm concentration during the initial flowering stage, or a dual spray application of 40 ppm concentration during the initial flowering stage and the rape pod stage, both resulted in a reduction of *S. sclerotiorum* incidence by more than 34% throughout the entire growth cycle of rapeseed. The primary mechanism underlying *S. sclerotiorum* inhibition is attributed to the effect of copper ions. Additionally, it is speculated that CM NFs have the effect of enhancing the immunity of rapeseed, which could be a potential reason for the reduction of incidence throughout the growth cycle of rapeseed when sprayed with CM NFs once or twice.

The good antifungal properties of CM are likely to be attributed to two factors: the increase in the surface area of nanoflakes resulting in the enhancement of the contacting probability between cupric ions and fungal cells; and the semiconductive physical properties of CM NFs facilitates the generation of ROS.

## Data Availability

The datasets used during the current study available from the corresponding author on reasonable request.
